# The Role of Endothelium in COVID-19

**DOI:** 10.3390/ijms222111920

**Published:** 2021-11-03

**Authors:** Mihaela Ionescu, Anca Pantea Stoian, Manfredi Rizzo, Dragos Serban, Domenico Nuzzo, Laura Mazilu, Andra Iulia Suceveanu, Ana Maria Dascalu, Irinel Raluca Parepa

**Affiliations:** 1 Cardiology Department, Faculty of Medicine, Ovidius University of Constanţa, 900527 Constanţa, Romania; ciucea.mihaela@yahoo.com (M.I.); irinel_parepa@yahoo.com (I.R.P.); 2 Diabetes, Nutrition, and Metabolic Diseases Department, Faculty of Medicine, Carol Davila University, 050474 Bucharest, Romania; ancastoian@yahoo.com (A.P.S.); manfredi.rizzo@unipa.it (M.R.); 3 Department of Health Promotion, Mother and Child Care, Internal Medicine and Medical Specialties, University of Palermo, 90100 Palermo, Italy; 4 Forth Surgery Department, Emergency University Hospital Bucharest and Faculty of Medicine, Carol Davila University, 050474 Bucharest, Romania; dragos.serban@umfcd.ro; 5 Italian National Research Council, Institute for Research and Biomedical Innovation (CNR-IRIB), 90100 Palermo, Italy; 6 Oncology Department, Faculty of Medicine, Ovidius University of Constanţa, 900527 Constanţa, Romania; lauragrigorov@gmail.com; 7 Internal Medicine Department, Faculty of Medicine, Ovidius University of Constanţa, 900527 Constanţa, Romania; andrasuceveanu@yahoo.com; 8 Department of Ophthalmology, Emergency University Hospital Bucharest and Faculty of Medicine, Carol Davila University, 050474 Bucharest, Romania; ana.dascalu@umfcd.ro

**Keywords:** endothelium, SARS-CoV-2, COVID-19, pathogenesis, complications

## Abstract

The 2019 novel coronavirus, known as severe acute respiratory syndrome-coronavirus 2 (SARS-CoV-2) or coronavirus disease 2019 (COVID-19), is causing a global pandemic. The virus primarily affects the upper and lower respiratory tracts and raises the risk of a variety of non-pulmonary consequences, the most severe and possibly fatal of which are cardiovascular problems. Data show that almost one-third of the patients with a moderate or severe form of COVID-19 had preexisting cardiovascular comorbidities such as diabetes mellitus, obesity, hypertension, heart failure, or coronary artery disease. SARS-CoV2 causes hyper inflammation, hypoxia, apoptosis, and a renin–angiotensin system imbalance in a variety of cell types, primarily endothelial cells. Profound endothelial dysfunction associated with COVID-19 can be the cause of impaired organ perfusion that may generate acute myocardial injury, renal failure, and a procoagulant state resulting in thromboembolic events. We discuss the most recent results on the involvement of endothelial dysfunction in the pathogenesis of COVID-19 in patients with cardiometabolic diseases in this review. We also provide insights on treatments that may reduce the severity of this viral infection.

## 1. Introduction

The inner cellular lining of arteries, veins, and capillaries is formed by the vascular endothelium, which is a continuous monolayer of endothelial cells. It functions as a barrier between tissues and blood in a mechanism that is similar to an endocrine organ. Through its dynamic interaction with blood components and other circulating cells, the endothelium is intimately engaged in many pathological processes. The diverse activities of this large endocrine organ are essential for maintaining hemostatic equilibrium under physiological circumstances [1].

The vascular endothelium is the crucial interface between blood and tissues. The endothelium presents numerous properties that contribute capitally to homeostasis. It displays a tightly regulated palette of functions that control vasomotion, vascular permeability, inflammation control, oxidative stress, and structure definition [2]. The endothelium has been disrupted in the pathophysiology of past coronavirus infections, either directly via signaling effects or indirectly through increased pro-inflammatory mediator synthesis and the consequent dysregulation of the coagulation cascade [3,4]. In general, endothelial dysfunction is caused by: (1) aging, (2) sex hormones and their decrease with age, (3) reactive oxygen species (ROS), (4) an increased ratio of circulating endothelium microparticles to progenitor cells (EMPs/PCs), and (5) a pro-inflammatory state [5,6].

SARS-CoV-2, the etiological agent of COVID-19, frequently produces clinical manifestations on multiple organ systems, particularly the lungs, brain, heart, and vasculature. Direct viral toxicity, endothelial cell damage and thrombo-inflammation, the dysregulation of the immune response, and the dysregulation of the renin–angiotensin–aldosterone system are all fundamental mechanisms that may play a role in the pathophysiology of multiorgan injury secondary to SARS-CoV-2 infection (RAAS). These pathways in COVID-19 pathophysiology are presently being studied to determine viral toxicity [7].

Acute hypoxic respiratory failure is the most common clinical symptom in COVID-19 patients, which often leads to acute respiratory distress syndrome (ARDS) and the requirement for invasive mechanical 3 ventilation [8,9].

Chronic deterioration of systemic endothelial function in patients with cardiovascular and metabolic diseases, compounded by the acute, noxious effects of SARS-CoV-2 on the endothelium, may explain patients’ worst COVID-19 symptoms. As a result, understanding the involvement of endothelial dysfunction in the pathogenesis of COVID-19 infection in patients with cardiometabolic diseases is important because it may provide a novel target for treatments that aim to reduce the severity of infection in this patient group. Endothelial dysfunction is also a frequent characteristic of major comorbidities that enhance the risk of SARS-CoV-2 infection, such as obesity, hypertension, diabetes, coronary artery disease, and heart failure [10].

## 2. Effects of Cytokine Storm in the Endothelial Cells

SARS-CoV2 causes hyper inflammation, hypoxia, apoptosis, and a renin–angiotensin system imbalance in a variety of cell types including macrophages, type II alveolar cells, T cells, and endothelial cells. Endothelial leak, cell death, systemic inflammation, and thrombosis may all be caused by high amounts of pro-inflammatory cytokines/chemokines. Endothelium with high levels of Ang-II adopts a pro-inflammatory and procoagulant character. Endothelial dysfunction may be caused by ARDS-induced hypoxia due to mitochondrial ROS production, intracellular acidosis, cell signaling pathway activation, and increased blood viscosity. Endothelial dysfunction and thrombosis are worsened by a dysregulated immunological response, hypercytokinemia, RAS imbalance, complement activation, and hypoxemia [11].

Pneumocytes, local macrophages, and dendritic cells, which are immediately targeted by the virus, are the first to generate chemokines and pro-inflammatory mediators in the infected area. Then, neutrophils and monocytes-macrophages (the main producers of pro-inflammatory cytokines) travel from the bloodstream to the inflammation site, where they enter alveolar or interstitial spaces and initiate the so-called “cytokine storm”. This causes further epithelial and EC damage. The excessive and unregulated release of pro-inflammatory cytokines spreads throughout the body via circulation, causing vascular permeability and leakage, coagulation activation, immune cell differentiation, and, finally, death in ARDS single or multiple organ failure [12].

Pro-inflammatory cytokines, such as IL-1 and TNF-alpha, stimulate gene expression in each other, creating an amplification loop that keeps the cytokine storm going. The endothelial cell is a major target of cytokines because they activate nuclear factor-B, which is a crucial pro-inflammatory transcriptional hub. IL-1 also induces significant increases in the synthesis of IL-6, which is the initiator of the hepatocyte acute phase response, by endothelial and other cells. Fibrinogen, the clot’s precursor, and PAI-1, the main inhibitor of our natural fibrinolytic system, are among the acute phase reactants. C-reactive protein (CRP), which is frequently high in COVID-19, is a simple biomarker for the inflammatory state. The acute phase response alters the thrombotic/fibrinolytic balance. This promotes thrombosis in the arteries, the microvasculature, organs such as the heart and kidneys, and the veins, which produces deep vein thrombosis and predisposes patients to pulmonary embolism. Thus, the same cytokines that induce aberrant endothelial function may also trigger the acute phase response, which, in combination with local endothelial dysfunction, can lead to COVID-19 clinical consequences [13].

Endothelial dysfunction is a significant predictor of future clinical occurrences [14]. The oxidation of small, dense low-density lipoproteins (LDLs), becoming oxidized LDLs in the arteries and accumulating as foam cells, plays a major role in the first step of the atherosclerotic process [15]. The predominance of small dense LDLs is strictly correlated with inflammatory cytokines [16], and these LDL subspecies are usually elevated in patients at high cardiovascular risk and in patients with specific ethnic backgrounds [17,18,19,20]. Small, dense LDLs are atherogenic because of greater arterial entry and retention than the larger, more buoyant counterparts. In addition, they have increased oxidative susceptibility (and, therefore, quickly become oxidized LDLs) as well as a reduced affinity for the LDL receptor (and, therefore, slowly circulate in the plasma) [21]. Small, dense LDLs have been recognized as an independent risk factor for cardiovascular diseases. Independently of the LDLs’ concentrations, these particles are a key factor for the development and progression of atherosclerosis [15,21]. They are, therefore, a target of therapy [22,23,24]. Notably, increasing evidence suggests that the qualities (particle size and functionality) of both HDL and LDLs impact cardiovascular risk beyond the quantity of such lipoproteins (plasma concentrations) [25,26].

The renin–angiotensin system (RAS) may be triggered by the inflammatory pathway induced by SARS-CoV-2, either directly by raising angiotensin I (Ang I) or indirectly by decreasing the surface expression of angiotensin-converting enzyme 2 (ACE2). ACE2 causes Ang II to be hydrolyzed into the vasodilator Ang (1–7), which inhibits Ang II’s effects and therefore counteracts activation of the RAS [27]. It is important to recognize that abnormal free radical production, a downstream consequence of cytokine storm, is the main cause of endothelial dysfunction, which leads to direct cell injury and various organ failure. The antiproliferative, antithrombotic, and antiatherogenic phenotype is maintained by NO, which is generated by endothelial nitric oxide 5 synthase (eNOS), which is a critical factor for vascular homeostasis. Endothelial dysfunction and thrombotic events are characterized by a decrease in NO bioavailability as a consequence of decreased NO generation and/or enhanced NO breakdown by ROS. Low levels of the metabolite Ang-(1–7), which, under physiological circumstances, exerts an essential antioxidative and vaso-protective function via a strong increase in NO, are determined by reduced ACE2 activity [27], as shown in COVID-19.

The endothelial gateway selectively controls endothelial permeability and promotes vascular integrity under physiological conditions. A myriad of systems, including the vascular endothelial-cadherin (VE-cadherin, CD144), is required for an intact endothelial barrier [28]. The integrity of this single-cell layer that separates the blood compartment from the tissues may be jeopardized by a variety of problems. Endothelial cell sloughing and death may be induced by a variety of processes, including pyroptosis and apoptosis, when endothelial viability is compromised. Pro-inflammatory cytokines and reactive oxygen species are two triggers for these pathways of programmed cell death [29,30].

## 3. Proof of Endothelial Dysfunction in COVID-19

The presence of ACE2 on endothelial cells, smooth muscle cells, and perivascular pericytes in almost all organs indicates that SARS-CoV-2 may readily travel throughout the body once it enters the circulatory system [31]. In [31], the authors discovered higher numbers of ACE-2-positive endothelial cells as well as significant changes in endothelial morphology, including cell swelling, disruption of intercellular junctions, and cell death, in post-mortem lung tissues from patients who died from COVID-19 or acute respiratory distress syndrome caused by influenza A (H1N1) infection, compared to age-matched, uninfected control lungs.

Systemic hypertension, diabetes, and obesity, in which endothelial dysfunction is known to be a major factor, are the most frequent comorbidities seen in COVID-19 patients that are linked with a poorer prognosis and a greater incidence of mortality [32]. The presence of viral inclusion structures in endothelial cells has also been shown, indicating that these cells have been directly infected by the virus; pathologic examination of these specimens showed endotheliitis of the submucosal arteries [33]. The authors hypothesize that the presence of viral components in the endothelium attracts immune cells through the ACE2 receptor, resulting in widespread endothelial dysfunction and death. In a similar vein, an autopsy case series revealed significant pulmonary endothelial damage linked to the intracellular virus and disturbed cell membranes [34]. A histologic examination of pulmonary arteries revealed extensive thrombosis with microangiopathy; alveolar-capillary microthrombi were nine times more common in COVID-19 patients than in influenza patients.

Another study looked at skin and lung tissues from COVID-19 patients and found that the pattern of COVID-19 pneumonitis was mostly pauci-inflammatory septal capillary damage. Significant deposits of terminal complement components including C5b-9 (membrane assault complex), C4d, and MBL-associated serine protease 2 (MASP-2) in the microvasculature followed [35]. This is in line with the theory that COVID-19 infection results in a catastrophic microvascular damage syndrome, which includes complement pathway activation, endotheliitis, and a thrombotic condition [36]. In humans, the SARS-CoV-2 cell entry receptor ACE2 and the entry-associated receptor TMPRSS2 are expressed together in a variety of tissues and cell types [37]. Endothelial cells in human blood arteries and the microvasculature have been shown to express ACE2. TMPRSS2 expression has been found in the human vascular endothelium [38,39], but investigations are limited. Angiotensin-converting enzyme (ACE) is a major regulator of the renin–angiotensin system, which regulates hemodynamic homeostasis in the body [40]. ACE2 is an analog of ACE. The ACE/ACE2 ratio seems to be important in maintaining the RAS, and its dysregulation has been linked to arterial thrombosis [41,42]. An ACE/ACE2 balance is also important for maintaining endothelial integrity in arteries, which may lead to thrombosis if it is disrupted [36]. Although no specific evidence for SARS-CoV-2 exists at this time, in vitro studies for SARS-CoV-1 and HCoV-NL63 binding on ACE2 in lung tissue indicate that virus binding downregulates ACE2 (but not ACE). This causes an ACE/ACE2 imbalance and promotes tissue injury, which may activate prothrombotic cascades inside the vessels [43].

**Figure 1:**
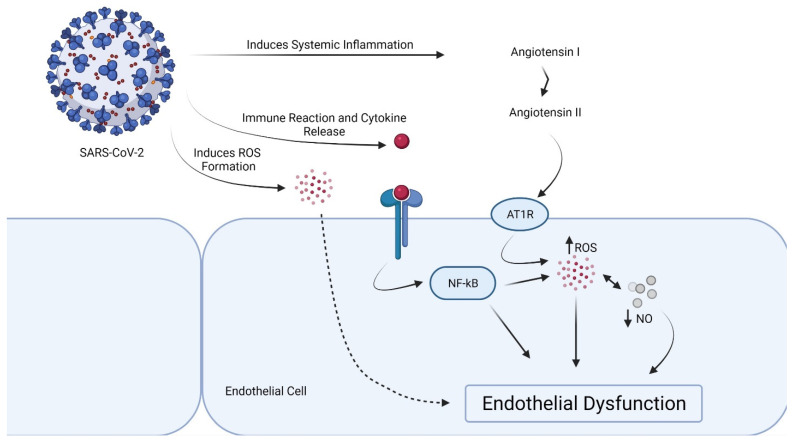
Proposed mechanism of endothelial dysfunction in SARS-CoV-2 infection.

By accelerating the breakdown of angiotensin II to angiotensin 1–7, a lower ACE/ACE2 ratio in the vascular endothelium inhibits the initiation of the prothrombotic cascade. Angiotensin 1–7 binds to G-protein coupled Mas receptors and has antithrombotic properties. A greater ACE/ACE2 ratio, on the other hand, allows angiotensin II to bind to AT1 receptors, causing vasoconstriction, inflammation, and ultimately thrombosis [44]. SARS-CoV-2-mediated ACE2 downregulation in vascular endothelium may also activate the kallikrein–bradykinin pathway, leading to platelet aggregation and vessel leakage, which can lead to thrombosis [45] (Figure 1). Notably, the endothelial dysfunction can be provoked by a subunit of SARS-CoV-2 spike protein, the spike protein subunit 1, without an intact virus [46].

## 4. Local Dysregulation of the RAS and Endothelial Dysfunction

Hypertension, cardiovascular disease, diabetes, and obesity are the most prevalent comorbidities in COVID-19 patients [47,48,49,50]. COVID-19 individuals with no known comorbidities had a crude mortality rate of 0.9 percent, compared to 10.5 percent in patients with cardiovascular disease and 7.3 percent in those with diabetes [50]. Furthermore, these comorbidities seem to be strongly linked to age, which appears to be the most powerful predictor of COVID-19-related mortality. When compared to a lower age group, those aged 45 and above are more likely to die from COVID-19. The vasculature undergoes complicated structural and functional changes as people age, resulting in endothelial and smooth muscle cell dysfunction. Endothelial cells’ capacity to generate NO and react to agonist and mechanical stimuli is substantially decreased as they age [51]. Given the importance of endothelial dysfunction in the pathogenesis of hypertension, cardiovascular disease, diabetes, and obesity, investigating the involvement of endothelial dysfunction in the etiology of COVID-19 may provide important insights. Furthermore, new evidence suggests that SARS-CoV-2 may cause vascular damage, implying that pre-existing endothelial dysfunction coupled with SARS-direct CoV-2’s attack on the vascular system may explain the increased mortality of COVID-19 patients with comorbid conditions [33,34].

Dysregulation of the local RAS system caused by the SARS-CoV-2 infection may be one mechanistic reason for these clinical findings. The RAS system’s components have been found in organs such as the heart, lungs, and liver, which operate via autocrine and paracrine processes that are independent of circulating RAS [52,53]. In the injury/repair response, inflammation, fibrogenesis pathways, and the organ-based RAS system play key roles. In an acute lung injury model produced by acid aspiration, for example, mice lacking ACE2 had substantially increased pulmonary vascular permeability, which is a characteristic of acute lung injury/ARDS in people [54]. As a result of SARS-CoV-2 binding to ACE2 and the downregulation of ACE2, the loss of ACE2 protective functions in the lung’s local RAS system, which is independent of the continuing viral infection, is anticipated.

**Figure 2:**
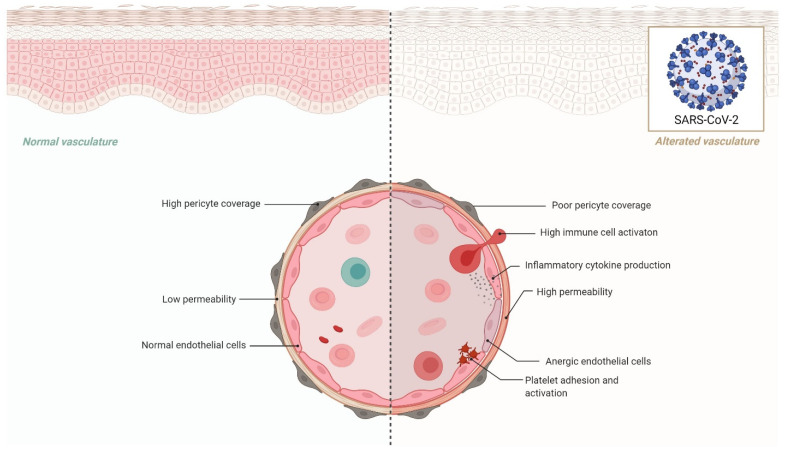
Effects of endothelial dysfunction in COVID-19.

Another important tissue-specific RAS organ is the heart. In rats, administration of an ACE2 activator (e.g., Diminazene aceturate) has been shown to reduce ischemia-induced heart damage, increase circulating endothelial progenitor cells, and restore the RAS system’s average balance. The autopsies of SARS patients revealed viral RNA and decreased expression of ACE2 in the heart [55], which may explain the observed cardiac damage in COVID-19 cases. According to recent research, SARS-CoV-2 may predispose COVID-19 patients to cardiac injuries owing to the loss of cardioprotective function of ACE2, or patients with heart failure have a greater chance of SARS-CoV-2 infection and consequent cardiac damages [56]. These findings indicate that SARS-CoV-2 may pose several challenges to the circulatory system, as well as the pulmonary and cardiac vasculature, via altering ACE2 activity. Although mechanistic investigations are required in this setting to identify high-risk people and create possible treatments, alternative pathways through the circulatory system and other target organs are also required [57] (Figure 2).

## 5. Heart Failure and COVID-19

In a variety of ways, cardiovascular diseases (CVD) may be linked to increased susceptibility to COVID-19 and/or the severity of consequences. Subjects with pre-existing heart failure (HF) or HF risk factors are more likely to develop HF as a consequence of any viral infection. To obtain entrance to the epithelium, SARS-CoV-2 utilizes ACE2, which is produced by lung epithelial cells, as the receptor-binding domain for its spike protein [58]. In HF, ACE2 (and ACE) gene expression is increased [59,60]. This may potentially increase infection susceptibility. Studies have also found that medications used for hypertension, diabetes, and heart failure (HF)—such as ACE inhibitors and angiotensin receptor blockers (ARBs)—may cause ACE2 upregulation (even at the cardiac level), thereby increasing susceptibility to infection [60,61,62]. However, there is currently no evidence for this, and the American College of Cardiology, American Heart Association, Heart Failure Society of America, and British Cardiovascular Society have all recommended that people should not stop taking these medicines [63,64].

Heart failure (HF) was reported by Zhou et al. in 23% of hospitalized COVID-19 patients, and it was more prevalent in patients who did not survive COVID-19 compared to patients who did survive (51.9% vs. 11.7%, *p* < 0.001) [65]. The high HF prevalence among COVID- 19 patients might be related to the failure of preexisting cardiomyopathy with impaired left ventricular function, or it might be a consequence of a COVID-19 related myocardial injury, both through direct and immune-mediated mechanisms. Right heart failure with increased pulmonary vascular resistance and pulmonary hypertension should also be considered, especially in the context of moderate-severe pneumonia and ARDS [66]. The coexistence of HF and COVID-19 complicates diagnosis and management. However, there are significant differences in the chest CT scans of patients with HF compared to the chest CT scans of patients who have HF and COVID-19, such as the enlargement of pulmonary veins lesions distribution and morphology [67].

Patients with HF have a higher risk of venous thromboembolism due to preexisting risk factors such as blood stasis in the legs, heart disease, and endothelial damage [68,69]. Patients with ischemic cardiomyopathy and atrial fibrillation, on the other hand, are at risk for arterial thrombosis [68]. Oral contraceptives, hormone replacement treatment, and breast cancer are all extra thrombotic hazards in women with HF [70]. The consequences of COVID-19 infection on the risk of stroke or pump thrombosis in patients with long-term LVAD support who are already at high risk for thrombosis are presently unclear. Unless there is a significant contraindication, therapeutic medications should be maintained in patients with HF who are on anticoagulant medication and need to be admitted for COVID-19. Anticoagulant prophylaxis should be given to all hospitalized HF patients with COVID-19 infection who do not have a preexisting condition [71]. In individuals with HF, underlying pulmonary illness is prevalent. Approximately 30% of patients with HF had chronic obstructive pulmonary disease, which raises their risk of hospitalization and death [72]. In addition to increased left ventricular filling pressures, these individuals may develop pulmonary hypertension as a result of parenchymal lung illness [73].

Hypoxemic respiratory failure and ARDS may increase pulmonary vasoconstriction and interstitial edema in patients with COVID-19 infection, increasing pulmonary hypertension in individuals without pre-existing lung illness [74]. Further elevations in pulmonary pressures due to ARDS may impair right ventricular function in individuals who already have biventricular failure. Patients with advanced HF, particularly those on long-term LVAD support, have significantly decreased functional capacity as assessed by peak VO2, as well as an impaired ability to increase cardiac output in response to physiological stresses. These 11 variables, taken together, reduce their cardiac reserve [75,76].

Both acute and chronic HF is caused by systemic inflammation. The production of pro-inflammatory cytokines, such as tumor necrosis factor-alpha, interleukin-6, and interleukin-1 beta, is stimulated by the hemodynamic stress of HF. Furthermore, concomitant comorbidities such as obesity, type 2 diabetes, and hypertension may prolong an inflammatory state, resulting in multiorgan involvement and endothelial dysfunction. C-reactive protein, lactate dehydrogenase, N-terminal pro–B-type natriuretic peptide, and interleukin-6, which are used to evaluate COVID-19 severity, may already be increased in HF patients, including patients on LVAD support. Therefore, data acquired in the COVID-19 setting should be compared to starting values [76,77,78,79].

Despite attempts to prevent transmission through physical distancing and other measures, COVID-19 is spreading across neighborhoods. Patients with HF who self-isolate are also at risk of contracting COVID-19 because they are often exposed to caregivers. Advanced care planning is essential for all patients with HF, including all populations on LVAD support and HT recipients, especially for patients in regions where COVID-19 infection is common [80]. Clinicians should start these discussions with patients and their caregivers as soon as they are diagnosed with HF, rather than waiting until they are admitted to the hospital. Care teams should include HF experts in discussions on the objectives of care, including the deactivation of implanted cardioverter-defibrillators for patients with HF who develop COVID-19 and need hospital admission.

## 6. Future Perspectives

COVID-19 patients frequently have clinical manifestations of multiple organ systems, particularly the lungs, brain, heart, kidney and vasculature, and there is already evidence of some post- COVID manifestations [81,82]. Considering all available evidence, we can conclude that endothelial dysfunction plays an important role in the pathogenesis of COVID-19, particularly for patients with pre-existing comorbidities such as diabetes, obesity, hypertension, or cardiovascular disease. We know that these patients have a higher risk of developing more severe symptoms and complications when infected by SARS-CoV-2 [83,84,85]. In addition, the presence of these comorbidities is associated with a greater need for intensive care during COVID-19 and related mortality [86,87,88]. Numerous studies have shown that broadly used treatment with statins, renin–angiotensin system inhibitors, or beta-blockers improve endothelial function and organ-associated complications, although the duration of therapy is an important factor [89]. Thus, we cannot exclude that such drugs may be helpful to prevent some complications of SARS-CoV2 infection, as long as there are no contraindications for these treatments.

## 7. Conclusions

In this article, we discussed how endothelial dysfunction associated with COVID-19 can cause impaired organ perfusion that may generate acute myocardial injury, renal failure, and a procoagulant state resulting in thromboembolic events. Providing these insights on the molecular mechanisms of COVID-19 can help improve the management and treatment of patients. This gives us new hope during this terrible pandemic.
